# A cognitive modeling approach to learning and using reference biases in language

**DOI:** 10.3389/frai.2022.933504

**Published:** 2022-11-16

**Authors:** Abigail G. Toth, Petra Hendriks, Niels A. Taatgen, Jacolien van Rij

**Affiliations:** ^1^Bernoulli Institute for Mathematics, Computer Science and Artificial Intelligence, University of Groningen, Groningen, Netherlands; ^2^Center for Language and Cognition Groningen, University of Groningen, Groningen, Netherlands

**Keywords:** cognitive modeling, implicit causality, language learning, PRIMs cognitive architecture, predictive processing, reference biases

## Abstract

During real-time language processing, people rely on linguistic and non-linguistic biases to anticipate upcoming linguistic input. One of these linguistic biases is known as the *implicit causality bias*, wherein language users anticipate that certain entities will be rementioned in the discourse based on the entity's particular role in an expressed causal event. For example, when language users encounter a sentence like “Elizabeth congratulated Tina…” during real-time language processing, they seemingly anticipate that the discourse will continue about Tina, the object referent, rather than Elizabeth, the subject referent. However, it is often unclear how these reference biases are acquired and how exactly they get used during real-time language processing. In order to investigate these questions, we developed a reference learning model within the PRIMs cognitive architecture that simulated the process of predicting upcoming discourse referents and their linguistic forms. Crucially, across the linguistic input the model was presented with, there were asymmetries with respect to how the discourse continued. By utilizing the learning mechanisms of the PRIMs architecture, the model was able to optimize its predictions, ultimately leading to biased model behavior. More specifically, following subject-biased implicit causality verbs the model was more likely to predict that the discourse would continue about the subject referent, whereas following object-biased implicit causality verbs the model was more likely to predict that the discourse would continue about the object referent. In a similar fashion, the model was more likely to predict that subject referent continuations would be in the form of a pronoun, whereas object referent continuations would be in the form of a proper name. These learned biases were also shown to generalize to novel contexts in which either the verb or the subject and object referents were new. The results of the present study demonstrate that seemingly complex linguistic behavior can be explained by cognitively plausible domain-general learning mechanisms. This study has implications for psycholinguistic accounts of predictive language processing and language learning, as well as for theories of implicit causality and reference processing.

## 1. Introduction

Events in the world typically do not occur in a random fashion. For example, dark clouds in the sky often precede rain. And we, as humans, can use our knowledge about the non-randomness of events in order to make predictions about future events. For example, if we see dark clouds in the sky, we can predict that there will be rain, which may lead to us grabbing an umbrella when leaving the house. When we use our past experiences in order to anticipate upcoming events, we are engaging in what is known as predictive (or anticipatory) processing. Predictive processing is widely assumed to be a core aspect of human cognition and has been shown across various cognitive domains such as executive functioning, motor coordination, and visual perception (Clark, 2013; see Bubic et al., 2010 for a review of the literature on predictive processing in cognition). Furthermore, predictive processing is not a particular form of processing that only gets utilized in special circumstances, but rather is the general means by which we humans facilitate and optimize normal processing in terms of speed and accuracy (LaBerge, 2013).

Language processing is one aspect of cognition where predictive processing plays a particularly crucial role. Given the rapid speed (~2 words per second in spoken conversational English) and pervasive ambiguity of language, it is not always possible for language users to wait until all relevant information is received before making a decision about the interpretation of an utterance; and instead, language users must be able to anticipate upcoming linguistic input. Although the exact role and nature of anticipation is a topic of debate in the psycholinguistic literature, there is accumulating evidence that language users indeed generate predictions about upcoming input (see Kuperberg and Jaeger, 2016; Pickering and Gambi, 2018 for reviews of the literature on prediction in language comprehension). Perhaps the most well-known example comes from the influential visual world eye-tracking study by Altmann and Kamide (1999). In that study, participants listened to sentences like “the boy will eat/move the cake,” while viewing scenes containing various objects, with only one object being edible (i.e., a cake). The results showed that in the “eat” condition, participants fixated on the cake in the visual scene before actually hearing the word “cake,” which was not the case in the “move” condition. This finding was suggested to be driven by the fact that after hearing the verb “eat,” participants could anticipate that the direct object would be something edible, and because the cake was the only one edible object in the visual scene, they could anticipate hearing the word “cake.”

In order for input to be predictable, there crucially must be a systematic relationship between the current state and (potential) upcoming states. In situations where such a systematic relationship exists, language users may have a preference for one potential continuation over all the possible continuations. Such a bias is what leads language users to anticipate certain upcoming linguistic input. There is evidence that language users use all sorts of biases in order to anticipate upcoming input during real-time language comprehension. Such biases include semantic biases (e.g., Altmann and Kamide, 1999; Grisoni et al., 2017), syntactic biases (e.g., Wicha et al., 2004; Van Berkum et al., 2005; Otten et al., 2007), and lexical form biases (e.g., DeLong et al., 2005; Dikker et al., 2010; Ito et al., 2018). For example, in the Altmann and Kamide (1999) study, participants used a lexical-semantic bias in order to predict that something edible would follow “eat.”

Despite the evidence that language users use biases in order to anticipate upcoming linguistic input, we still know very little about how the biases are acquired and how exactly they get used during real-time language processing. There are various reasons for this. Firstly, although biases are likely to be picked up from asymmetries in the linguistic input, this assumption often goes untested due to the difficulty of approximating actual language input in its context of use. Second, the limited number of acquisition studies that do exist tend to focus on answering questions like *at what age do children do/use x*? and such questions do not necessarily tell us anything about *how* exactly something is acquired. Finally, language comprehension does not have a physical reflection. Therefore, it cannot be directly observed (as can be done with speech and language production) and instead it must be measured. Measures of real-time language comprehension require a linking hypothesis between what is being measured and what is happening in terms of cognitive and brain processes. Such linking hypotheses are often not fully explicit in terms of all the intermediate steps of getting from point A to point B. For example, in visual world eye-tracking (Cooper, 1974; Eberhard et al., 1995; Allopenna et al., 1998), it is assumed that eye gaze is a reflection of where attention is focused and thus what is being processed in real-time. However, it is not clear what happens in between hearing the speech signal and fixating on certain entities.

The present study crucially aims to reveal *how* language users acquire and use biases during real-time language comprehension. We are able to address the limitations listed above by applying cognitive modeling to simulate both the learning and online use of biases. This allows us to assess learning in a controlled environment and also forces us to generate concrete hypotheses, where the entire process of getting from point A to point B must be made explicit. Determining how biases are acquired and how they are used during real-time language processing may be crucial for understanding the exact nature of adult patterns of language comprehension and may also yield cognitively plausible and testable predictions for children's acquisition and use of these biases. As such, the results of the present study will likely have implications for psycholinguistic theories about prediction and language learning, as well as for theories about the specific biases themselves. In the present study we will primarily focus on one type of bias, known as the implicit causality bias, which will be introduced in the following section.

## 2. Background

### 2.1. What is the implicit causality bias?

Implicit causality describes the preference to attribute the cause of particular events to certain entities. For example, when asked to make causality judgements about sentences like those in (1), participants consistently attribute the cause of the apologizing event in (1a) to Kaitlyn and the cause of the congratulating event in (1b) to Marie (e.g., Brown and Fish, 1983; Rudolph and Forsterling, 1997). In other words, in (1a) people are more likely to assume that Kaitlyn did something that warranted apologizing (e.g., forgetting to respond to an email), whereas in (1b) people are more likely to assume that Marie did something that warranted being congratulated (e.g., getting a job promotion).

(1) a. Kaitlyn apologized to Marie.
b. Kaitlyn congratulated Marie.

The verbs that describe these events are known as implicit causality verbs, which are further categorized depending on whether causality is attributed to the grammatical subject or the grammatical object, such that “apologize” is an example of a subject-biased implicit causality verb and “congratulate” is an example of an object-biased implicit causality verb. In the literature these verbs are also referred as “NP1-biased” and “NP2-biased” verbs, respectively. This has to do with the fact that, in English, the first noun phrase of a transitive sentence is the canonical position for grammatical subjects, with the second noun phrase being the canonical position for objects. However, we will stick with the “subject-biased” and “object-biased” terminology.

In addition to causality judgment tasks, evidence of implicit causality also comes from passage completion studies, where participants are presented with sentences like those in (2) and asked to complete the passage (e.g., Garvey and Caramazza, 1974; Stevenson et al., 1994; Kehler et al., 2008; Fukumura and Van Gompel, 2010; Rohde and Kehler, 2014).

(2) a. Molly frustrated Sophie.     _______________________      . (subject-biased)
b. Molly comforted Sophie.     _______________________     . (object-biased)

These studies looked at whether participants' completions begin by rementioning the subject referent or the object referent of the preceding clause. The findings consistently show that the distribution of the different completion types is highly dependent on the verb. That is, for sentences like (2a), participants are much more likely to remention Molly, the preceding subject referent, than Sophie, the preceding object referent, whereas the reverse is true for sentences like (2b). For other verbs, such as “filmed,” the distribution of rementioning the preceding subject referent vs. the preceding object referent is relatively equal. This clear preference to remention a certain referent for particular verbs seems to be driven by an implicit causality bias, such that participants prefer to continue with the causally implicated referent. That is, they prefer to remention the subject in (2a) *because* “frustrated” is a subject-biased verb, and they prefer to remention the object in (2b) *because* “comforted” is an object-biased verb. Furthermore, participants' completions for implicit causality verbs like “frustrated” and “comforted” are much more likely to be in the form of a causal explanation compared to more neutral verbs like “filmed,” highlighting the role of coherence relations (namely causal explanations) in implicit causality (see Kehler et al., 2008; Rohde et al., 2011). These findings have been taken as supportive evidence that implicit causality verbs are a special category of verbs and that within this category verbs differ with respect to which referent they causally implicate. Linguistic accounts of implicit causality generally assume that language users have somehow learned these different verb categories and use them when producing sentence completions (see Hartshorne, 2014 for a review of the different accounts of implicit causality). However, it is still an open question whether implicit causality information takes the form of hard verb categories or soft context-dependent preferences.

### 2.2. How is the implicit causality bias used during real-time language comprehension?

Critically for the present study, language users also seem to use their knowledge of implicit causality during real-time language comprehension (e.g., Van Berkum et al., 2007; Rohde et al., 2011; Järvikivi et al., 2017). For example, in a self-paced reading study Koornneef and Van Berkum (2006, Experiment 1) had participants read sentences like those in (3) (translated from Dutch), where “praise” is an object-biased implicit causality verb.

(3) a. David praised Linda because he had been able to complete the difficult assignment with her help only.
b. Linda praised David because he had been able to complete the difficult assignment with very little help.

The reaction time data revealed that participants were significantly slower to read sentences like (3a), where the gender of the pronoun in the because-clause was inconsistent with the bias set up by the verb in the main clause, compared to sentences like (3b) where the gender of the pronoun in the because-clause was consistent with the bias set up by the verb in the main clause. This main effect of verb type was significant immediately following the pronoun and was therefore taken as evidence that participants used their knowledge of implicit causality to anticipate which entity would get referred to. This finding was further supported by a follow-up eye-tracking study using the same materials (Koornneef and Van Berkum, 2006, Experiment 2).

Evidence for the influence of implicit causality during real-time language comprehension primarily comes from the pronoun resolution literature, which has typically been interested in identifying what sources of information get used to identify the antecedent of a pronominal form (e.g., “they,” “he,” “it”) (e.g., Arnold et al., 2000; Järvikivi et al., 2005; Cozijn et al., 2011; Garnham, 2013). For example, in a recent visual-world eye-tracking study, Kim and Grüter (2021) had participants listen to short stories about two referents that contained a critical pronoun, while simultaneously looking at pictures of the two referents. The authors manipulated whether the verb preceding the critical pronoun was subject-biased or object-biased. An example story can be seen in (4), with “like” being an object-biased implicit causality verb.

(4) Austin and Burt met at a cocktail party last week. Austin liked Burt right away because he really enjoyed hearing jokes.

The gaze data revealed that participants were more likely to fixate on the subject referent when the story contained a subject-biased verb than when it contained an object-biased verb, and similarly participants were more likely to fixate on the object referent when the story contained an object-biased verb than when it contained a subject-biased verb (see Pyykkönen and Järvikivi, 2010; Cozijn et al., 2011; Itzhak and Baum, 2015; Järvikivi et al., 2017 for similar findings). Thus, these pronoun resolution studies provide evidence that language users employ implicit causality information during real-time language comprehension.

Although these visual-world eye-tracking studies set out to investigate the influence of implicit causality on pronoun resolution, across the studies the effect of implicit causality was actually already found to arise before the onset of the pronoun and sometimes even before the causal connective (e.g., Pyykkönen and Järvikivi, 2010). This importantly suggests that participants were using implicit causality to proactively generate an expectation about which referent would get rementioned in the subsequent discourse (which is in line with the speculations of Koornneef and Van Berkum, 2006).

However, it is unclear how exactly implicit causality gets used during real-time language comprehension. It seems that whenever language users encounter an implicit causality verb, they generate an expectation that a causal explanation will ensue and that the causally implicated referent will be referred to. However, because online measures only serve as a proxy for the underlying cognitive processes, we cannot know exactly what is happening within the brain.

### 2.3. How is the implicit causality bias learned?

Despite the vast amount of research involving implicit causality, we know very little about how preferences related to implicit causality are acquired by language users. It is reasonable to assume that asymmetries between certain events and their causes exist in the world. For example, most people would agree that if you apologize to someone, most often it is because of something you did, whereas if you congratulate someone, it is most often because of something they did. That is, in the world, “apologizing” events are more likely to be caused by the *apologizer* than the *apologizee*, whereas “congratulating” events are more likely to be caused by the *congratultee* than the *congratulator*. And because people talk about the world they live in, similar asymmetries also likely exist in language input. For example, in a language user's linguistic input, subject-biased discourses are probably more likely to continue about the subject referent than the object referent, whereas object-biased discourses are probably more likely to continue about the object referent than the subject referent. If such asymmetries exist in the linguistic input, then language users could pick up on these asymmetries, which would result in an implicit causality bias.

Testing this assumption would require one to determine what the specific distributions are in real-world language input. This type of research is often carried out using large linguistic corpora, where tokens can be extracted and annotated by automatic parsing algorithms. Unfortunately, current algorithms are unable to extract and annotate the type of information that would be needed to determine specific discourse continuation distributions as they relate to implicit causality. This is because implicit causality deals with reference, where various different forms (e.g., “my neighbor,” “Steve,” “him”) can all be used to refer to a single entity, while at the same time, single forms (e.g., “he”) can be used refer to multiple entities (e.g., my neighbor, my dad, my brother). See Sukthanker et al. (2020) for a review on the difficulty that anaphora and co-reference pose for the field of natural language processing (NLP).

Given that automatic parsers are currently insufficient, Guan and Arnold (2021) recently carried out two small-scale corpus studies in order to investigate whether the implicit causality preferences of language users follow from frequent patterns of reference in natural language. In the first study the authors used Google to search for tokens that contained two animate pronouns and an implicit causality verb followed by the connective “because” (e.g., “he amazed me because”), which resulted in 548 tokens. They then hand-coded if the following clause rementioned the subject referent or the object referent (note that items could be coded as mentioning both the subject referent and the object referent). The results revealed that there was a strong tendency to remention causally implicated referents, such that subject referents were rementioned in 87% of the subject-biased verb tokens but only in 45% of the object-biased verb tokens, and similarly object referents were rementioned in 90% of the object-biased verb tokens but only in 53% of the subject-biased verb tokens. These findings support the hypothesis that language users pick up on reference asymmetries in the linguistic input. In the second corpus study, the authors used the Fisher Corpus (Cieri et al., 2004, 2005) of telephone conversations and did not find that causally implicated referents were more likely to be rementioned. However, in that second study, less restrictive sampling criteria were used, such that both animate and inanimate referents were sampled, and further the search was not limited by the presence of the connective “because.” This greatly influenced the content of the sampled tokens. For example, only ~10% of all the tokens contained a causal explanation, which was likely driven by the fact that most of the referents were inanimate and people are probably less likely to speak about inanimate entities causing events (see Nieuwland and Van Berkum, 2006 for a related discussion on animacy). This highlights the fact that natural language is rich and that you cannot only consider implicit causality in isolation but instead must consider how it interacts with all sorts of other information like animacy, gender, etc. To our knowledge, this paper by Guan and Arnold (2021) is the first and only to test the frequency-based account of implicit causality and as such provides a crucial first step to investigating how implicit causality is learned. Nevertheless, the study had the limitation of its relatively small sample sizes, such that it is unclear whether the pattern would hold across larger samples of naturalistic language. Furthermore, both the subject argument and the object argument of the tokens were restricted to pronominal forms, which could have skewed the results in systematic ways. More specifically, there is evidence that language users do not produce pronouns at equal rates for subject referents and object referents (see Arnold, 1998; Rohde and Kehler, 2014; Kehler and Rohde, 2019). For instance, in the sentence completion studies described above, as in example (2), participants were much more likely to remention subject referents using pronouns compared to using proper names, whereas participants were much more likely to remention object referents using proper names compared to using pronouns (Rohde and Kehler, 2014). In fact, this was the case regardless of the verb that was used (i.e., regardless of whether the verb was subject-biased or object-biased in terms of implicit causality), suggesting that pronoun production is insensitive to the implicit causality bias.

Despite the inconclusive findings, there is at least some evidence that implicit causality results from language users picking up on asymmetries in the linguistic input. However, even so, it is still unclear how exactly language users pick up on these asymmetries, i.e., what learning mechanisms are involved. The previous literature has tended to focus on answering questions concerning if and when implicit causality exerts its influence during language processing. Thus, we still know very little about how language users learn implicit causality, as well as how exactly it gets used when comprehending language in real-time.

## 3. Present study

The present study took a cognitive modeling approach and used computational simulations to investigate whether simple cognitive learning mechanisms could explain how language users learn implicit causality, as well as to explore how implicit causality may get used by language users during real-time language processing.

Cognitive modeling is a specific means of computationally simulating human cognition, where the aim is to construct cognitively plausible accounts of the phenomena in question. The primary goal of this subfield of artificial intelligence is to gain a better understanding of human cognition. Thus, importance is placed on being able to understand what exactly it is that the model is doing, and why. This differs from other branches of artificial intelligence, such as machine learning or deep learning, where the primary goal is to generate intelligent systems without the aim of modeling human cognition. Cognitive modeling is executed within a *cognitive architecture*, which not only serves as an interface for implementing models, but also, importantly, is a unified theory of cognition (i.e., specifies how the brain is organized and how information is processed). This type of modeling requires the modeler to specify exactly what information gets processed and, importantly, how it gets processed. Although cognitive modeling is used to investigate all sorts of cognitive processes, it has also specifically been applied to language contexts (e.g., Lewis and Vasishth, 2005; Van Rij et al., 2010; Reitter et al., 2011; Brasoveanu and Dotlacil, 2015; Vogelzang et al., 2021). Applying this method can help bridge the gap between classical linguistic approaches and domain general cognition, leading to more cognitively plausible and precise theories of language and communication, situated within general human cognition.

By utilizing cognitive modeling to investigate how language users learn implicit causality, as well as how language users employ implicit causality during real-time language comprehension, we are able to exert a level of experimental control that would otherwise not be possible. For example, we can completely control what knowledge the model (i.e., a simulated human) initially has, thereby eliminating the possibility that the observed behavior is actually driven by some unknown variable. Furthermore, because cognitive architectures are unified theories of cognition, they already contain domain-general learning mechanisms and thus, we can use these integrated mechanisms to determine whether the learning of linguistic biases can be explained by domain-general learning. Because the implicit causality bias is centered around the rementioning of certain referents, the present study also additionally investigated whether the same mechanisms could explain how language users learn and use referent form biases (i.e., the preference to remention subject referents using pronouns and the preference to remention object referents using names).

As such, we constructed a cognitive model and had it process sentences like “Eva congratulated Angela.” Upon receiving the sentence, the model made predictions about how the discourse would continue with respect to upcoming referents and their forms. Crucially, across all the discourse items the model was presented with, there were reference asymmetries. By making use of simple learning mechanisms within the cognitive architecture, the model should be able to optimize its behavior, resulting in biased behavior that is consistent with the asymmetrical input. Furthermore, after the learning period, we presented the model with a series of discourse items that were in some way novel (i.e., contained a novel verb, novel names, or both). This was done in order to investigate how any learned biases may generalize in new contexts.

In the next section we will begin by giving a brief overview of the cognitive architecture that we used. We will then describe the specific details of the reference learning model, including how learning was implemented. Then finally we will describe how we manipulated the linguistic input data that was presented to the model.

## 4. Methodology

### 4.1. PRIMs cognitive architecture

Although numerous different cognitive architectures exist (see Kotseruba and Tsotsos, 2020 for a review on cognitive architectures), similar language processing research has largely been carried out using the ACT-R cognitive architecture (Anderson et al., 1998; Anderson, 2007 and see Vogelzang et al., 2017 for a review of ACT-R cognitive models of language processing). We however, chose to implement our model using the more recently proposed *PRIMs* (*primitive information processing elements*) cognitive architecture (Taatgen, 2013, 2014), which was specifically adapted from ACT-R in order to address some of the limitations with respect to how information is learned, stored, and exchanged within ACT-R (see Taatgen, 2017, 2021 for details). PRIMs functions similar to ACT-R and is used to simulate how cognitive tasks are performed. A crucial difference is that in ACT-R all of the task-relevant information is typically provided to the model, whereas in PRIMs it is possible to simulate how task-relevant information is learned. This is because PRIMs was designed to handle bottom-up learning. Thus, PRIMs is more suited for the present study, which aims to investigate not only how biases are used during language processing but also, crucially, how they are being learned.

**Figure 1:**
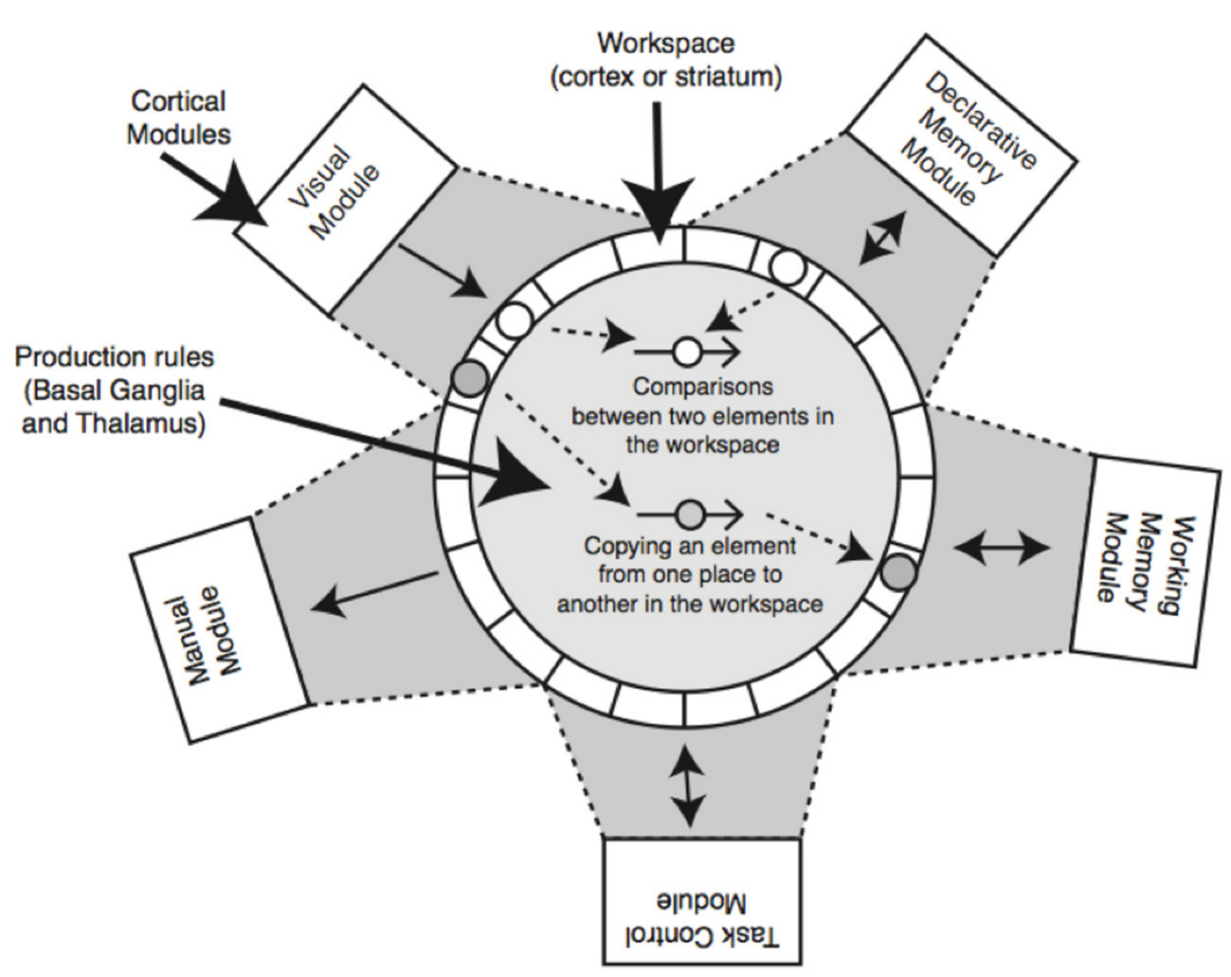
Visual representation of the PRIMs cognitive architecture. Taken from Taatgen (2013).

A visual representation of the PRIMs architecture is shown in Figure 1. Like in ACT-R and in many other cognitive architectures, cognition in PRIMs is modular, such that there are separate components for motor control, declarative memory, etc. In PRIMs, the separate modules interact with each other by exchanging information through their respective buffers, such that each module has its own buffer containing multiple slots that can each hold a piece of information. The buffers are able to exchange information through the use of operators (which function similar to production rules in ACT-R).

**Figure 2:**
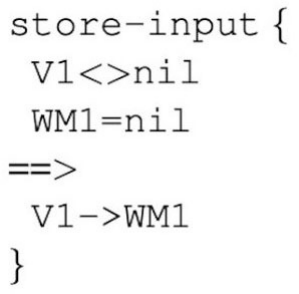
Example of a PRIMs operator. The “==>” arrow separates the condition prims from action prims. This operator copies information from slot 1 of the input buffer (V) into slot 1 of the working memory buffer (WM), as long as there is something in V1 and WM1 is empty.

Operators are built up from simple primitive elements, known as “prims,” which can perform simple actions, such as comparing two values or copy information. In order to avoid confusion, we will use uppercase notation “PRIMs” when referring to the cognitive architecture as a whole and lowercase notation “prims” when referring to primitive elements within the cognitive model. An example operator can be seen in Figure 2, where the “==>” arrow separates what are known as *condition prims* from *action prims*. The condition prims make simple comparisons between two different buffer slots (or determine if a certain buffer slot is empty), and the action prims move information from one buffer slot to another buffer slot.

In PRIMs, operators can either be learned bottom-up by having the model combine a series of prims or they can be pre-defined. In the present study we assume that language users have already learned certain language processing skills that can be further built upon through linguistic exposure, thus we opted to pre-define model-specific operators (i.e., operators that are needed to carry out the unique goals of the model). These pre-defined operators get stored as chunks in declarative memory and are retrieved on the basis of their activation. When an operator is retrieved, the model will check whether all the condition prims are satisfied. If so, then the model will carry out the action prims. If the condition prims are not satisfied, however, the action prims will not be carried out and the model will retrieve the operator with the next highest activation. Further features of the cognitive architecture are described below in relation to our specific model.

### 4.2. Our reference learning model

To characterize our reference learning model, we will discuss the separate stages that can be distinguished in how the model works, starting with sentence comprehension, followed by how the model generates predictions about upcoming referents and their forms, and ending with how the model learns from making predictions.

#### 4.2.1. Processing a transitive sentence

**Figure 3:**
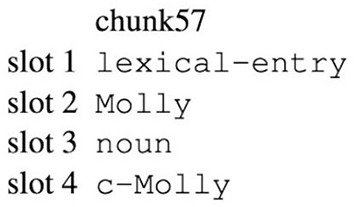
Example of a PRIMs lexical-entry chunk. The first line indicates the (arbitrary) chunk name. The second line indicates the type of chunk. The third line indicates the word form. The fourth line indicates the part of speech. The fifth line indicates the word's meaning, with “c” denoting “concept.”

First the model is presented with transitive sentence like “Leah fascinated Kathy” in its input buffer (denoted by an uppercase V), with each word taking up a single slot in the buffer (i.e., V1 = Leah, V2 = fascinated and V3 = Kathy). The model then processes each word one-by-one by retrieving an associated chunk from declarative memory. An example of a lexical-entry chunk can be seen in Figure 3, where the chunk contains information that is assumed to be associated with that particular lexical entry, such as its part of speech and semantic meaning. After the model retrieves the appropriate chunk, it copies the word into the working memory buffer (WM). The operators responsible for processing the first word are presented in Table 1 with descriptive detail. The model repeats this process for each word of the transitive sentence, which results in an event representation of the sentence being held in working memory. Because the buffer slots in PRIMs do not have labels, the order matters, thus for the simple transitive sentences studied in our particular model WM1 is always used to store information about the subject, WM2 is always used to store information about the verb, and WM3 is always used to store information about the object.

**Table 1:** Operators responsible for processing initial transitive sentence.

**Operator**	**PRIMs**	**Description**
retrieve-V1	V1 < >nil	Slot 1 of the input buffer (V) is not empty
	RT1=nil	Slot 1 of the retrieval buffer (RT) is empty
	WM1=nil	Slot 1 of the working memory buffer (WM) is empty
	==>	
	lexical-entry->RT1	Retrieve a ‘lexical-entry' chunk (from declarative memory),
	V1->RT2	with the additional specification that slot 2 of the chunk matches the information currently in V1
store-V1	V1=RT2	Slot 1 of the input buffer is the same as slot 2 of the retrieval buffer
	WM1=nil	Slot 1 of working memory is empty
	==>	
	RT2->WM1	Copy the information in slot 2 of the retrieval buffer into slot 1 of working memory

#### 4.2.2. Predicting the next referent

Once the model has processed the transitive sentence, it then predicts whether the discourse will continue about the subject referent (e.g., Leah) or the object referent (e.g., Kathy) of that sentence. In our model there is one operator that predicts the subject referent and another operator that predicts the object referent, both of which are presented in Table 2 with descriptive detail. Crucially the predict-subj operator copies information from WM1 (the subject of the transitive sentence) into WM5 (the slot used to store information about the subsequent referent), whereas the predict-obj operator instead copies information from WM3 (the object of the transitive sentence) into WM5.

**Table 2:** Operators responsible for predicting continued referent.

**Operator**	**PRIMs**	**Description**
predict-subj	WM3 < >nil	Slot 3 of working memory is not empty
	WM4=nil	Slot 4 of working memory is empty
	WM5=nil	Slot 5 of working memory is empty
	==>	
	WM1->WM5	Copy the information in slot 1 of working memory into slot 5 of working memory
predict-obj	WM3 < >nil	Slot 3 of working memory is not empty
	WM4=nil	Slot 4 of working memory is empty
	WM5=nil	Slot 5 of working memory is empty
	==>	
	WM3->WM5	Copy the information in slot 3 of working memory into slot 5 of working memory

#### 4.2.3. Predicting the next referent form

Once the model has predicted whether the discourse will continue about the subject referent or object referent of the transitive sentence, it then predicts whether that referent will take the form of a proper name (e.g., “Leah”/“Kathy”) or a pronoun (“she”). The operators responsible for this are presented in Table 3 with descriptive detail. The first two operators are highly similar, with one being used to predict a proper name when the model predicted that the next referent would be the subject referent and the other being used to predict a proper name when the model predicted that the next referent would be the object referent. The third operator in Table 3, predict-pro, is used to predict a pronoun both when the model predicted that the next referent would be the subject and when the model predicted that the next referent would be the object. The final operator in Table 3, retrieve-pro, will be retrieved by the model after the model carries out the action prims of the predict-pro operator. This was included in order to account for the fact that different pronouns could in principle be used to refer to different entities depending on gender and number agreement. However, only female referents were used in the input data in our study and therefore the only pronoun that gets used is “she.” It can also be seen that the predict-subj-name, predict-obj-name, and retrieve-pro operators all contain a “read-next -> AC1” prim. This action prim triggers the presentation of the subsequent discourse. This results in the model being presented with the actual discourse continuation in its input buffer (i.e., V1 = she and V2 = Leah), where the first slot is the form and the second slot is its meaning.

**Table 3:** Operators responsible for predicting continued referent form.

**Operator**	**PRIMs**	**Description**
predict-subj-name	WM4=nil	Slot 4 of working memory is empty
	WM1=WM5	Slot 1 and slot 5 of working memory are the same
	RT1=nil	The retrieval buffer is empty
	==>	
	WM5->M4	Copy the information in slot 5 of working memory into slot 4 of working memory
	read-next->AC1	Perform ‘read-next' action
predict-obj-name	WM4=nil	Slot 4 of working memory is empty
	WM3=WM5	Slot 3 and slot 5 of working memory are the same
	RT1=nil	The retrieval buffer is empty
	==>	
	WM5->WM4	Copy the information in slot 5 of working memory into slot 4 of working memory
	read-next->AC1	Perform ‘read-next' action
predict-pro	WM4=nil	Slot 4 of working memory is empty
	WM5 < >nil	Slot 5 of working memory is not empty
	RT1=nil	The retrieval buffer is empty
	==>	
	lexical-entry->RT1	Retrieve a ‘lexical-entry' chunk from the declarative,
	pronoun->RT3	with the additional specification that slot 3 of the chunk is ‘pronoun'
retrieve-pro	WM4=nil	Slot 4 of working memory is empty
	WM5 < >nil	Slot 5 of working memory is not empty
	RT1 < >nil	The retrieval buffer is not empty
	==>	
	RT2->WM4	Copy the information in slot 2 of the retrieval buffer into slot 4 of working memory
	read-next->AC1	Perform ‘read-next' action

Next, the model compares its predictions (stored in WM4 and WM5) to the newly presented input. If both the predicted referent (subject referent vs. object referent) and the predicted form of the referent (name vs. pronoun) match the input, then the model is issued a reward. In cases where the model predicted either the referent, the form of the referent, or both incorrectly, no reward is issued and the model must revise the contents of working memory to align with the actual subsequent discourse (as opposed to the predicted subsequent discourse). The operators responsible for this revision are presented in Table 4.

**Table 4:** Operators responsible for revising working memory.

**Operator**	**PRIMs**	**Description**
correct-re	WM4 < >V1	Slot 4 of working memory is not the same as slot 1 of the input buffer
	V3=nil	Slot 3 of the input buffer is empty
	==>	
	V1->WM4	Copy the information from slot 1 of the input buffer into slot 4 of working memory
correct-ref	WM5 < >V2	Slot 5 of working memory is not the same as slot 2 of the input buffer
	V3=nil	Slot 3 of the input buffer is empty
	==>	
	V2->WM5	Copy the information from slot 2 of the input buffer into slot 5 of working memory

#### 4.2.4. Learning mechanism within the reference learning model

Crucial to our research aims, we utilized PRIMs' *context-operator learning*. Context-operator learning is a mechanism for learning associative strengths and is implemented in the architecture as a type of reinforcement learning, such that whenever a reward is issued, the associative strengths between the current context and all of the operators that led to the reward being issued are increased. In PRIMs, “context” can refer to the entire global workspace (i.e., all of the slots of all the separate module buffers). However, for our particular model we were only interested in increasing the associative strengths between the operators and the slots in the working memory buffer. Thus, in our “Leah fascinated Kathy” example, if the model predicted that the discourse would continue about the subject referent Leah using the “she” pronoun, and this turned out to be the case (resulting in a reward), then the associative strengths between WM1 = Leah and all of the operators that were successfully retrieved up until that point (e.g., predict-subj, predict-pro, etc) would be increased, as would the associative strengths between WM2 = fascinated and WM3 = Kathy and all of those operators. The associative strengths between the working memory slots and all of the operators that were *not* retrieved remain unchanged.

This means that in similar contexts in the future, for example when WM2 = fascinated, the model will be more likely to retrieve the previously successful operators, given that operators are retrieved on the basis of their activation. However, in its initial state, before the model has processed a certain amount of input items (and updated the associative strengths), it is as equally as likely to fire certain sets of operators. Take, for example, the two operators predict-subj and predict-obj (Table 2). These two operators have the exact same condition prims and thus in a naive model the two operators are just as likely to fire. The same is true for the set of operators responsible for predicting the referent form.

Furthermore, if the distribution between the different possible discourse continuations is symmetrical in the input data, then the associative strengths between competing operators will cancel each other out and the model will retrieve the operators at chance. For example, if across all the “fascinated” discourses the model processes, the likelihood of continuing about the subject referent vs. the object referent is equal, then the associative strengths between WM2 = fascinated and predict-subj and between WM2 = fascinated and predict-obj will be equal and therefore the two operators will be just as likely to be retrieved. However, if the distribution between the different possible discourse continuations is *not* symmetrical, then the associative strength for one of the competing operators will eventually be higher, making that operator more likely to be retrieved.

Thus, if there are asymmetries in the input data, *via* prediction and reinforcement learning our model can learn which combination of operators is most likely to lead to a reward given the current context. As such, we constructed asymmetrical input data to present to our model with the aim of investigating how asymmetries in the input influence learning.

### 4.3. Input data

The input data consisted of 10,000 unique items that were presented to the model in two parts. Part 1 was a simple transitive sentence, consisting of a verb with its subject and object arguments, each in the form of a proper name. Part 2 was a discourse continuation, referring to either the subject referent or the object referent of the transitive sentence, either in the form of a proper name or a pronoun. This means that in total there were four different item types, which are illustrated in (5).

(5) a. Ashley repulsed Sarah. Ashley
b. Ashley repulsed Sarah. She (= Ashley)
c. Ashley repulsed Sarah. Sarah
d. Ashley repulsed Sarah. She (= Sarah)

Each item was uniquely created by sampling from 10 different verbs and 40 randomly generated female names. The transitive sentence was generated by sampling one verb (from the list of 10) and two names (from the list of 40), where one name was for the subject argument and the other was for the object argument. The 10 verbs were chosen from the Ferstl et al. (2011) sentence completion corpus, which was conducted in order to obtain implicit causality norming measures. In line with our critical assumption that asymmetries in linguistic input drive reference biases, we opted to select an unequal number of the different implicit causality verb types. As such, we selected five subject-biased verbs (repulsed, angered, fascinated, disappointed and apologized), three object-biased verbs (comforted, feared, and congratulated), and two more or less neutral verbs (interrupted and filmed). Because we do not know what the actual distribution is in English, this is simply one possible distribution, which could be compared to other possible distributions (see the Discussion for further commentary).

**Figure 4:**
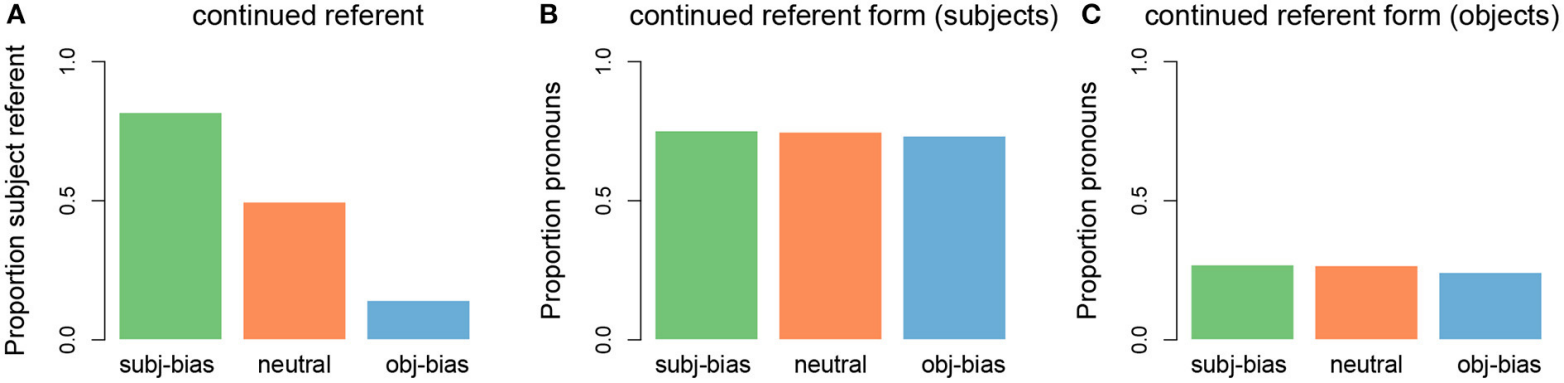
Distributions of discourse continuations in the input data. **(A)** Proportion of subject referent continuations for each implicit causality verb type (green: subject-biased, orange: neutral, blue: object-biased). **(B)** Proportion of pronouns for subject referent continuations for each verb type. **(C)** Proportion of pronouns for object referent continuations for each verb type.

The discourse continuation was generated based on two unique sampling probabilities, one relating to the next referent (subject vs. object of the first sentence) and one relating to the next referent form (proper name vs. pronoun). For the continued referent, we converted the implicit causality measures obtained in the Ferstl et al. (2011) corpus, into sampling probabilities. For example, in the corpus “repulsed” had an implicit causality score of 76 (meaning that 76% of all subject/object continuations were about the subject), therefore for all of our “repulsed” items the sampling probability of the continued referent being the subject vs. the object was 0.76/0.24. Thus, each of the 10 verbs had a unique sampling probability. With respect to the continued referent form, we opted for a general pronoun bias for subject continuations (where the probability of sampling a pronoun was 0.75) and a general name bias for object continuations (where the probability of sampling a name was 0.75). This was inspired by the sentence completion literature, which has shown that the rates of pronominalization are not affected by implicit causality, with instead only position and/or grammatical role modulating the use of pronouns (e.g., Kehler and Rohde, 2019; but see Weatherford and Arnold, 2021 for alternative findings). Thus, the cycle of generating a single item was as follows: 1) sample a verb and two names (subject and object arguments) to create the transitive sentence, 2) sample either the subject referent or the object referent of the transitive sentence (using verb-dependent probability) to be the next referent, and 3) sample either a name or a pronoun (using grammatical position-dependent probabilities) to be the next referent form. This was repeated 10,000 times. The distributions of the constructed input data can be seen in Figure 4. It should be noted that because we used sampling probabilities, the percentages are not always exact. In other words, a sampling probability of 0.75 does not always result in an outcome of exactly 75%, which is why the different bars in Figures 4B,C have slightly different heights.

The model was presented with all 10,000 items from the input data in a completely randomized order. For each item the model 1) processed the transitive sentence, 2) predicted whether the next referent would be the grammatical subject or grammatical object of the transitive sentence, and then 3) predicted whether the next referent form would be a proper name or a pronoun. The model was then presented with the actual discourse continuation. In cases where the model's predictions matched the continued discourse, the model was issued a reward. In cases where the model's predictions did not match the continued discourse, it would 4) additionally update the contents of working memory to match the continued discourse. In order to eliminate any order effects, we ran the model 100 separate times, where each run consisted of the model being presented all 10,000 items in different randomized order. Each of these 100 model runs essentially simulates a different language learner starting without any prediction biases. This allowed for us to analyze the average behavior of a group of (simulated) language learners.

Additionally, at the end of each run, we presented the model with a series of items that were in some way novel. This was done in order to further explore the outcome of the learning and determine if any learned biases would generalize in new contexts. This series of items was comprised of 1) five completely novel items, where the transitive sentence verb and both its subject and object arguments had not appeared in the input data, 2) five novel verb items, where the transitive sentence verb had not appeared in the input data, but both its subject and object arguments had and 3) fifteen novel name items, where the transitive sentence verb had appeared in the input data (five subject-biased, five object-biased, and five neutral), but both its subject and object arguments had not.

In the next section we will begin by examining how the model's predictions about the next referent changed as the model was presented with an increasing amount of input. We will then examine how the model's predictions about the next referent form changed as the model was presented with an increasing amount of input. Finally, we will look at how the model dealt with novel input data and examine the generalized biases that the model was able to learn.

## 5. Results

### 5.1. Predicted next referent

**Figure 5:**
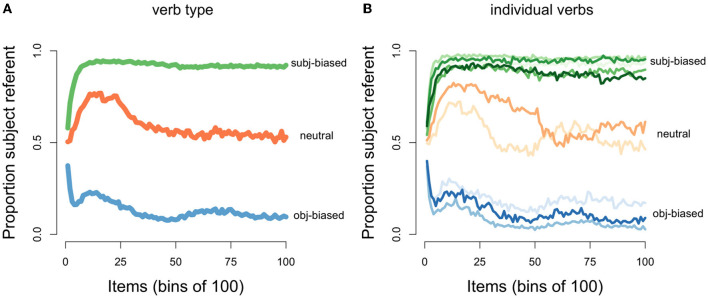
Grand average proportions of subject referent continuation predictions (x-axis) by the total number of presented input items (y-axis; binned into bins of 100 items). **(A)** Separated out for each implicit causality verb type (green: subject-biased, orange: neutral, blue: object-biased). **(B)** Separated out for for each implicit causality verb (greens: subject-biased verbs, oranges: neutral verbs, blues: object-biased verbs).

After the model processed a transitive sentence, it made a prediction about the next referent, namely whether the next referent would be the subject or the object referent of the transitive sentence. We were interested in how the model's predictions about the next referent would change as the model was presented with an increasing amount of input for each of the three verb types (subject-biased verbs, object-biased verbs, and neutral verbs). This pattern can be seen in Figure 5A, which shows how the proportion of predicting that the next referent would be the subject referent changed as the number of presented input items increased, separated by verb type and averaged over the 100 model runs (where each model run simulates one participant). For the initial items, the model predicted that the next referent would be the subject referent at around chance level, across all three verb types. However, as the model was presented with an increasing amount of input, the proportion of predicting that the next referent would be the subject referent uniquely changed for each verb type. For subject-biased implicit causality verbs (green line), the proportion of predicting that the next referent would be the subject referent steadily increased, reaching a ceiling after ~1,200 items (12 bins of 100). For object-biased implicit causality verbs (blue line), the proportion of predicting that the next referent would be the subject referent steadily decreased for the first ~500 items, after which subject predictions slightly increased for the next ~500 items, before again decreasing and leveling off after ~4,000 items. For the implicit causality neutral verbs (orange line), the proportion of predicting that the next referent would be the subject referent initially increased, although not as steeply as for the subject-biased verbs, and then after ~1,500 items there was a shallow decrease, before leveling off at ~5,000 items. Note that in all cases where the next referent was not predicted to be the subject referent, the next referent was predicted to be the object referent, and vice versa. These results are in line with our expectations that the model's predictions would reflect the input data. As can be seen in Figure 4A, in the input data subject-biased items primarily continued about the subject referent, object-biased items primarily continued about the object referent, and neutral items were at chance.

We also wanted to examine how the model's predictions about the next referent would change for each individual verb within the three verb categories. Recall that our input data consisted of five unique subject-biased verbs, three unique object-biased verbs, and two unique implicit-causality-neutral verbs. This can be seen in Figure 5B, which shows that the verbs within the same category cluster together but that each verb still has a unique pattern. It can also be seen that between the two neutral verbs, there is quite a bit of variation in how the model's predictions about the next referent changed as the model was presented with an increasing amount of input.

### 5.2. Predicted next referent form

**Figure 6:**
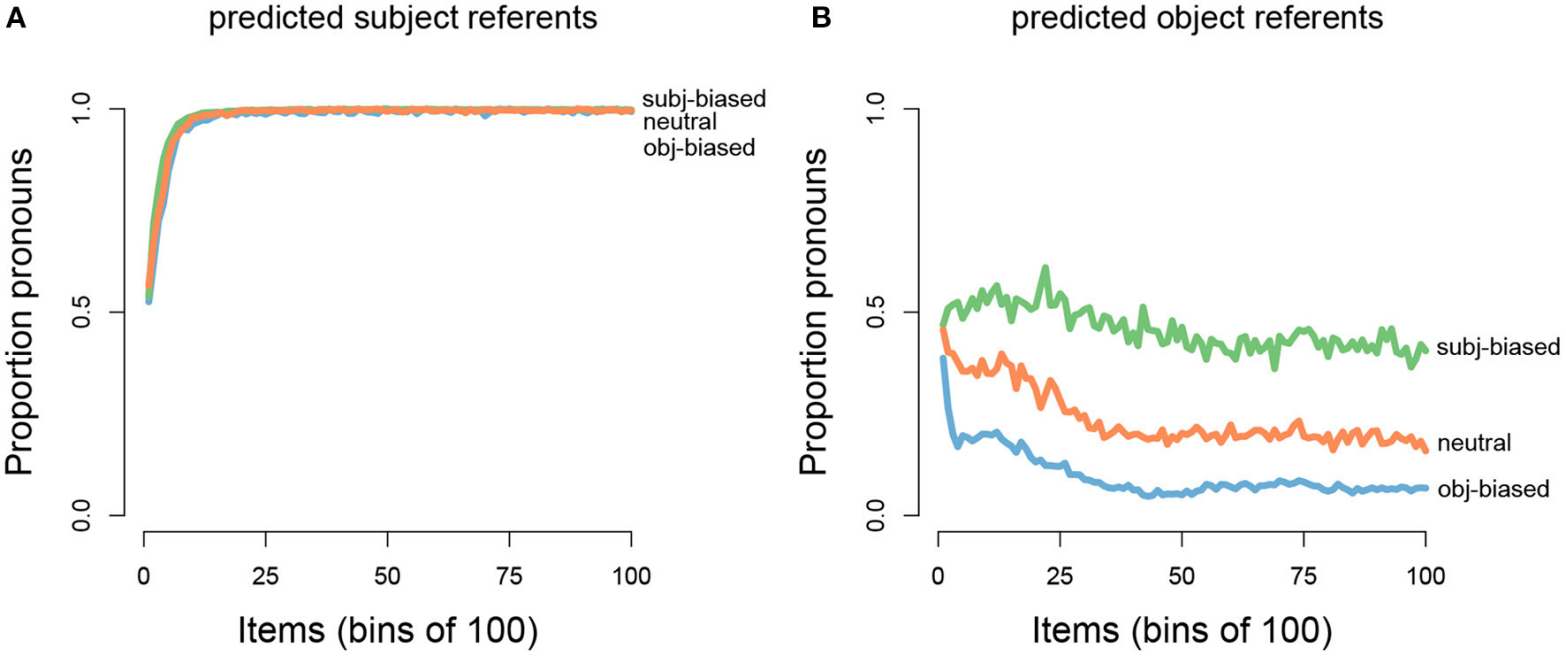
Grand average proportions of pronoun predictions (x-axis) by the total number of input items (y-axis; binned into bins of 100 items) for each implicit causality verb type (green: subject-biased, orange: neutral, blue: object-biased) for **(A)** subject referent continuations and **(B)** object referent continuations.

After the model predicted the next referent, it then further predicted the form of the next referent, namely whether the next referent form would be a proper name or a pronoun. We were interested in how the model's predictions about the next referent form would change as the model was presented with an increasing amount of input for each of the three verb types. This pattern can be seen in Figure 6, which shows the proportion of predicting that the next referent would be in the form of a pronoun, separated out depending on whether the model predicted the next referent to be the subject referent (left panel) or the object referent (right panel) of the transitive sentence and for each verb type (again averaged over 100 model runs, where each model run simulates one participant). It can be seen that in cases where the model predicted the next referent to be the subject, the proportion of pronoun predictions steadily increased for all three verb types, reaching a ceiling after ~1,500 items. In cases where the model predicted the next referent to be the object, pronoun predictions gradually decreased for all three verb types, however there was an interaction such that pronoun predictions decreased at different rates for each verb type: For subject-biased verbs (green line) the decrease was very gradual and leveled off at about 40% pronoun predictions, whereas for object-biased verbs (blue line) the decrease was much steeper (especially for the very early items) and leveled off at about 10% pronoun predictions. For the neutral verbs (orange line) the pattern was somewhere in between. Note that in all cases where the next referent form was not predicted to be a pronoun, it was predicted to be a proper name, and vice versa. In sum, the model picked up on the main trends in the input data, showing a pronoun bias for predicted subject referents and a name bias for predicted object referents. However, we also found a seemingly asymmetrical effect of implicit causality on referent form predictions, such that implicit causality influenced predicted object referent continuations but not predicted subject referent continuations.

**Figure 7:**
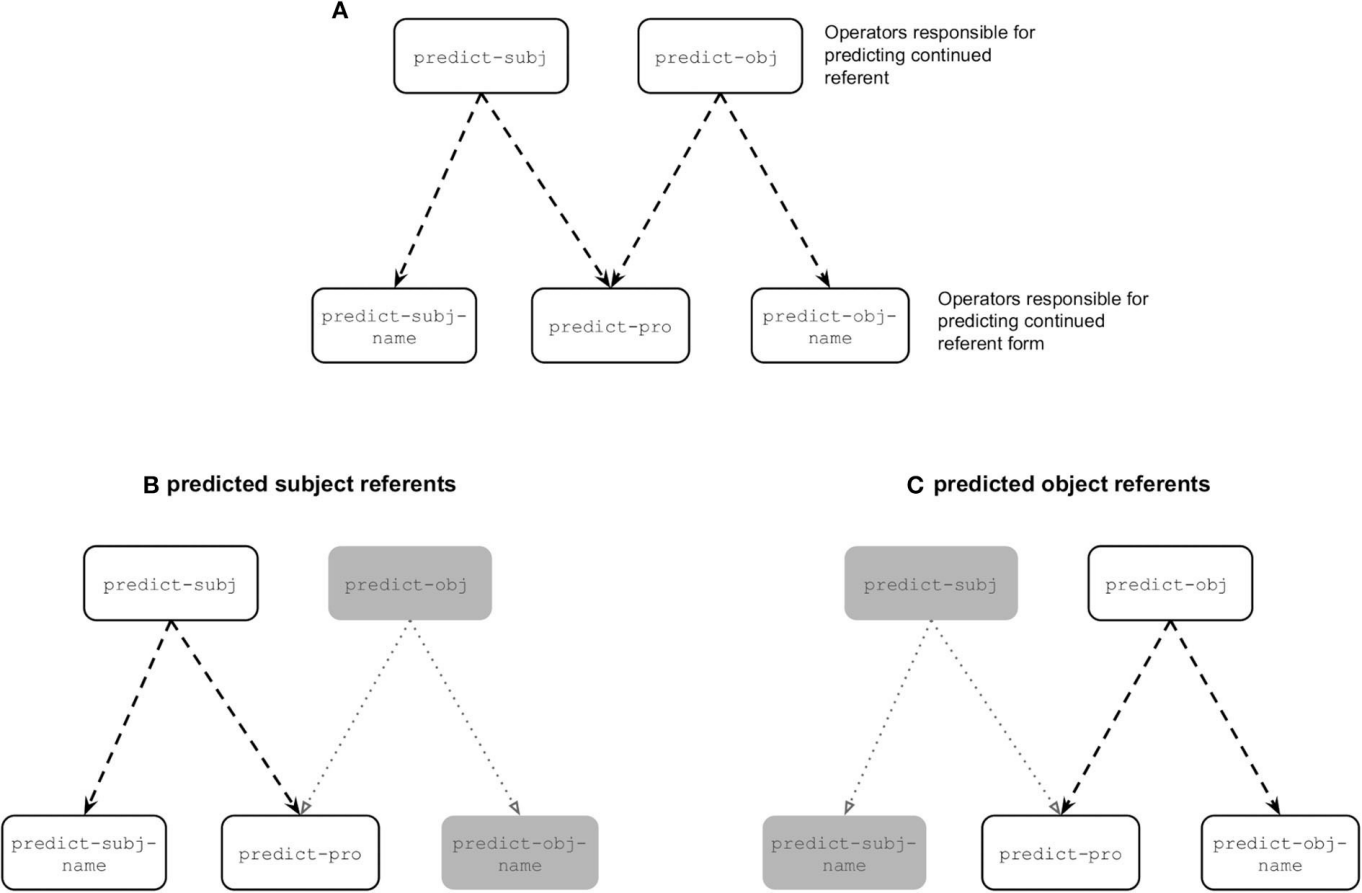
**(A)** Visual representation of all the operators responsible for predicting both the referent and referent form. The bottom two panels depict the two competing operators in cases where the model predicted the continued referent to be the subject referent **(B)** and in cases where the model predicted the next referent to be the object referent **(C)**, where the grayed out boxes represent the operators that are unable to be carried out in the given scenario.

First, we consider the main trends. Figure 7A illustrates the operators responsible for predicting both the continued referent and continued referent form. In cases where the model predicted the next referent to be the subject referent there was competition between two form operators, namely predict-subj-name and predict-pro (see Figure 7B). The competition between these two operators ended up being biased toward the predict-pro operator because in the input data subject referents were largely referred to using pronouns (see Figure 4B). In cases where the model predicted the next referent to be the object referent there was also competition between two form operators, this time predict-obj-name and predict-pro (see Figure 7C). The competition between these two operators ended up being biased toward predict-obj-name because in the input data object referents were largely referred to using names (see Figure 4C). This explains why we see a pronoun bias for predicted subject referents and a name bias for predicted object referents.

However, two aspects of these referent form predictions warrant further discussion. First, the influence of implicit causality on referent form predictions is surprising under the assumption that the model predictions should reflect the input data. As can be seen in Figure 4C, in the input data there was no difference in the amount of pronouns used to refer to object referents across the three verb types, yet we see a difference in the model's predictions. Second, it is also interesting that such an influence of implicit causality would only affect object referent predictions but not subject referent predictions. We will begin by describing how implicit causality ended up influencing referent form predictions and then why it seemingly only affected form predictions in the case of predicted object referents.

Our model inspection revealed that the reason implicit causality influenced referent form predictions has to do with the fact that the individual verbs ended up having positive associations with one of the operators responsible for predicting referent form. For example, subject-biased verbs ended up having a strong positive association with the predict-pro operator and object-biased verbs ended up having a strong positive association with the predict-obj-name operator. These positive associations are the result of rewards being issued in cases where the model correctly predicted both the referent and the referent form. This increased the associative strengths between the current context - including the verb - and all of the operators that led to the reward being issued - including the the referent form operator. So essentially, subject-biased verbs ended up having a strong positive association with the predict-pro operator because of all the times the model successfully selected the predict-pro operator (predominantly after a subject referent prediction) and object-biased verbs ended up having a strong positive association with the predict-obj-name operator because of all the times the model successfully selected the predict-obj-name operator (predominantly after an object referent prediction).

The reason we see an interaction between implicit causality and referent form predictions in case of predicted object referents is because these verb-specific associations interact with the general bias to predict names for object referents. For example, in the rare cases where the model predicted an object continuation for a subject-biased verb, the verb specific-bias toward the predict-pro operator competes with the general bias toward the predict-obj-name operator, which results in these two competing operators being relatively equal in terms of activation. This leads to the pattern seen in Figure 6B, where the likelihood of predicting a name vs. a pronoun is almost equal for subject-biased verbs.

These verb-specific associations in theory should also affect referent form predictions in cases where the model predicted a subject referent continuation. For example, in the rare cases where the model predicted a subject continuation for an object-biased verb, the verb-specific association should interact with the general bias to predict pronouns for subject referents. The reason this does not end up being the case is because object-biased verbs have a positive association with the predict-obj-name operator, however the condition checking of that operator (namely that the predicted referent is an object) means that the operator cannot fire in cases where the model predicted the continued referent to be the subject (hence it being grayed out in Figure 7B). Thus, the condition checking eliminates the possibility of there being competition between the verb-specific bias toward the predict-obj-name operator and the general bias toward the predict-pro operator, and as such prevents implicit causality from having an influence on referent form predictions in the case of predicted subject referents.

To summarize, the model picked up on the main trends in the input data. However, implicit causality influenced referent form predictions in the case of predicted object referent continuations, which does not align with the input data. Model inspection revealed that this was driven by competition between verb-specific associations with operators responsible for predicting referent form and general referent form biases. Furthermore, a similar interaction would have been found in the case of predicted subject referents, however the condition checking of the relevant operator prevented this interaction from surfacing. Whether our model's prediction that implicit causality will have an influence on online predictions about upcoming referent form is empirically correct or not remains an open question and needs to be tested against experimental data with human language users.

### 5.3. Novel items

Based on the model's predictions about the next referent and its form, it is clear that the model was able to pick up on the asymmetries that were present across the 10,000 input items the model was presented with. In order to further assess these learned biases and examine how they generalize, we presented the model with a series of items that had the same structure as the original input items but for which the transitive sentence was in some way novel. We were interested in what predictions the model would make with respect to the next referent and next referent form for these novel items. In order to ensure that each item was equally novel, we turned off the learning mechanism.

#### 5.3.1. Predicted next referent

**Figure 8:**
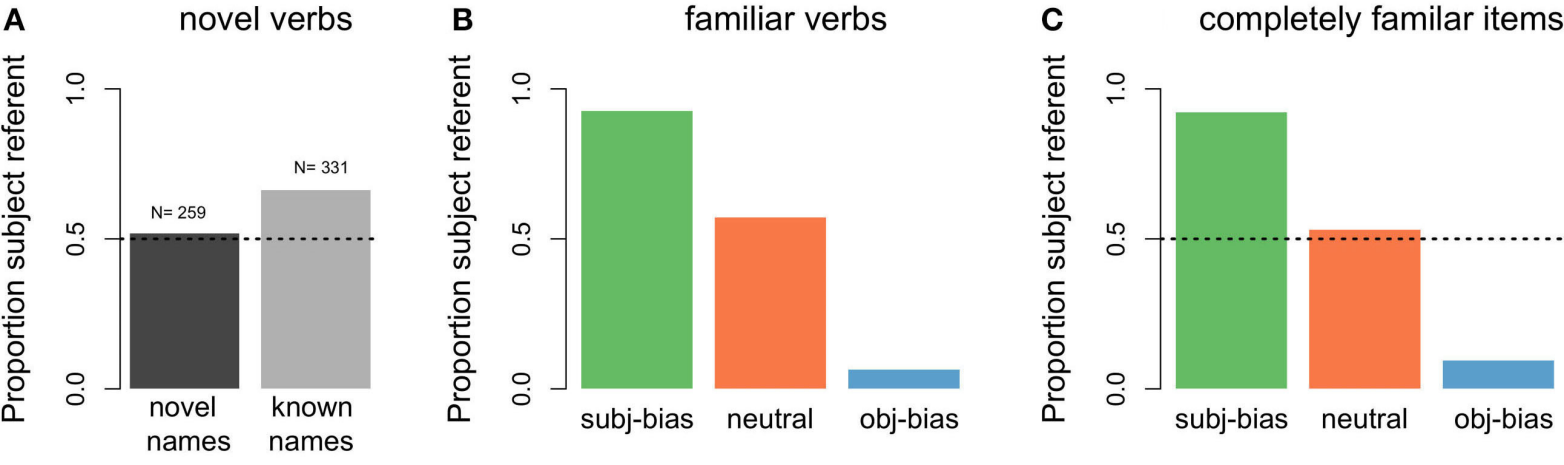
Proportion of predicted subject referent continuations by novelty status. **(A)** Items that contained a novel verb (dark gray: items that additionally contained novel subject and object arguments, light gray: items for which the subject and object arguments were familiar to the model). **(B)** Items that contained a familiar verb (green: subject-biased, orange: neutral, blue: object-biased) but for which the subject and object arguments were novel. **(C)** Items that contained a familiar verb and familiar subject and object arguments (these were the final 100 training input items, included for comparison purposes).

With respect to the model's predictions about the next referent, Figures 8A,B show the proportion of predicting that the next referent would be the subject referent for the different novel items. For comparison purposes, in Figure 8C we also present the predictions of the model for the final 100 items of the original 10,000 input items, which reflect the predictions the model has learned to make by the end of the learning phase. As can be seen in Figure 8A, for items that were completely novel (both the transitive sentence verb and its subject and object arguments), the model was at chance level for predicting that the next referent would be the subject referent. For items where the transitive verb was novel, but its subject and object arguments were familiar to the model (meaning the names appeared in the original input items), the model was above chance for predicting that the next referent would be the subject referent. Thus, the model learned a generalized subject referent continuation bias, reflecting that (simulated) language users are more likely to assume that discourses will continue about the subject referent as compared to the object referent, in the absence of any verb specific information.

As can be seen in Figure 8B, for items where the verb was familiar to the model (meaning the verb appeared in the original input items), but its subject and object arguments were novel, the proportion that the model predicted that the next referent would be the subject referent depended on the implicit causality of the verb: For subject-biased verbs the model was almost at ceiling for predicting that the next referent would be the subject referent, for object-biased verbs the model was near floor for predicting that the next referent would be the subject referent, and for neutral verbs the model made subject predictions slightly above chance. Thus, the model also learned a generalized implicit causality bias, such that when there is an implicit causality verb, (simulated) language users assume that the discourse will continue about the causally implicated referent. These predictions are in line with the final model predictions for completely known items (see Figure 8C) and reflect the proportional distributions of subject referent continuations in the original input data (see Figure 4A). The main conclusion of these next referent predictions for novel items is that there is a baseline subject referent continuation bias that gets modulated by a verb specific implicit causality bias (as is clearly illustrated by the familiar neutral verbs, where the model predicted that the next referent would be the subject referent at greater than chance level, but still lower than the baseline subject referent continuation bias).

#### 5.3.2. Predicted next referent form

**Figure 9:**
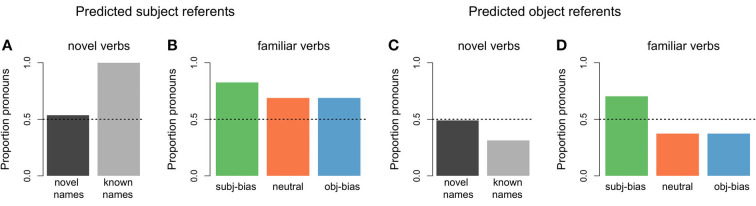
Proportions of predicted pronouns by novel item type. **(A)** Items that contained a novel verb where the model predicted a subject referent continuation (dark gray: items that additionally contained novel subject and object arguments, light gray: items for which the subject and object arguments were familiar to the model). **(B)** Items that contained a familiar verb but for which the subject and object arguments were novel, where the model predicted a subject referent continuation (green: subject-biased, orange: neutral, blue: object-biased). **(C)** Items that contained a novel verb where the model predicted an object referent continuation. **(D)** Items that contained a familiar verb but for which the subject and object arguments were novel, where the model predicted a object referent continuation.

With respect to the model's predictions about the form of the next referent, Figure 9 shows the proportion of predicting a pronoun across the different types of novel items, for both predicted subject referent continuations and predicted object referent continuations. For items that were completely novel (i.e., both the transitive verb and its subject and object arguments), the model was at chance level for predicting that the next referent form would be a pronoun. This was the case for both predicted subject referent continuations (dark gray bar in Figure 9A) and predicted object referent continuations (dark gray bar in Figure 9C). For items where the transitive verb was novel, but its subject and object arguments were familiar to the model (meaning the names appeared in the original input items), the proportion of predicting that the next referent form would be a pronoun largely depended on whether the model had predicted the next referent to be the subject referent vs. the object referent: When the model predicted a subject referent continuation, it almost exclusively predicted that the form would be a pronoun (light gray bar in Figure 9A). When the model predicted an object referent continuation, it was below chance for predicting that the form would be a pronoun, instead showing a preference for a proper name (light gray bar in Figure 9C). Thus, the model learned two distinct form biases, such that (simulated) language users are more likely to assume that continued subject referents will be in the form of pronouns, whereas continued object referents will be in the form of proper names, in the absence of any verb-specific information.

For items where the verb was familiar to the model (meaning the verb appeared in the original input items), but its subject and object arguments were novel, the proportion of predicting that the next referent form would be a pronoun depended on both the type of verb and whether the model had predicted the next referent to be the subject referent vs. the object referent: When the model predicted a subject referent continuation, the proportion of predicting a pronoun was above chance for all three verb conditions, but even more so for subject-biased implicit causality verbs (Figure 9B). When the model predicted an object referent continuation, the proportion of predicting a pronoun was still above chance for subject-biased implicit causality verbs (although lower than for predicted subject continuations), but below chance for both object-biased implicit causality verbs and neutral verbs (Figure 9D). This finding that pronoun predictions were partially influenced by verb type, and more specifically that subject-biased verbs showed a pronoun bias even in cases where the model predicted an object referent continuation, is unexpected given that in the input data there was no difference in the amount of pronouns used to refer to subject referents between the three verb types, nor was there a difference in the amount of pronouns used to refer to object referents between the three verb types (again see Figures 4B,C). This again has to do with the operator condition checking in the model.

## 6. Discussion

The aim of the present study was to gain a better understanding of reference biases in language, both in terms of how they are learned and how they are used during real-time language comprehension. Using the PRIMs cognitive architecture (Taatgen, 2013, 2014), we developed a reference learning model that simulated the learning of next mention referent biases (i.e., expectations that particular discourses will continue by rementioning certain referents) and rementioned referent form biases (i.e., expectations that particular rementioned referents will be expressed using a particular form), while at the same time simulating how these biases may get used during real-time language comprehension. More specifically, the implemented model processed sentences like “Nicole apologized to Sarah” and then predicted 1) whether the next mentioned referent would be the subject referent (i.e., Nicole) or the object referent (i.e., Sarah) and then 2) whether the predicted next referent form would be a proper name (i.e., “Nicole”/“Sarah”) or a pronoun (i.e., “she”). Across the input data that the model was presented with there were asymmetries in terms of which referent the discourse would continue about (i.e., after subject-biased implicit causality verbs there were more subject referent continuations and after object-biased implicit causality verbs there were more object referent continuations), as well as the form certain referents would take (i.e., continued subject referents were more often referred to using a pronoun and continued object referents were more often referred to using a proper name). These reference asymmetries were inspired by patterns found in the sentence completion literature based on studies of mostly English (e.g., Ferstl et al., 2011; Rohde and Kehler, 2014; Kehler and Rohde, 2019).

With respect to the model's predictions about which referent the discourse would continue with (i.e., the subject referent vs. the object referent of the preceding transitive sentence), during the initial items when the model was still naive to the next mention asymmetries present in the input, the model was equally as likely to predict that the discourse would continue about the subject referent as the object referent across all implicit causality verb types (subject-biased, object-biased, and neutral). Crucially, as the model was presented with increasing amounts of input, it became more likely to predict that the discourse would continue about the subject referent after subject-biased implicit causality verbs and less likely to predict that the discourse would continue about the subject referent after object-biased implicit causality verbs. Thus, the model's predictions about which referent the discourse would continue about ended up reflecting the asymmetries in the input data, indicating that an implicit causality bias was learned.

With respect to the model's predictions about which form the continued referent would take (i.e., a proper name vs. a pronoun), during the initial items when the model was naive to the form asymmetries present in the input, it was equally as likely to predict that the form of the next referent would be a proper name as a pronoun, for both predicted subject referents and object referents. As the model was presented with increasing amounts of input, in general it became more likely to predict that rementioned subject referents would take the form of a pronoun and less likely to predict that rementioned object referents would take the form of a pronoun. These findings are expected given that in the input data subject referents were predominantly referred to using pronouns, whereas object referents were predominantly referred to using names. Thus, the model was able to pick up on the main trends in the data, indicating that two different form biases were learned.

However, we also found a seemingly asymmetrical effect of implicit causality on referent form predictions, such that implicit causality influenced predicted object referent continuations but not predicted subject referent continuations. It was revealed that the reason implicit causality influenced referent form predictions was because the individual verbs ended up having positive associations with specific form operators. For example, the subject-biased verb “apologized” had a positive association with the predict-pro operator. Thus, in the rare cases where the model predicted an object referent continuation for this subject-biased verb, there was competition between the general bias to predict names for object referents and the verb specific bias to predict a pronoun. This interaction ultimately resulted in an increase in the proportion of pronoun predictions for subject-biased verbs (compared to object-biased verbs) when the model predicted an object referent continuation. The reason we did not see an influence of implicit causality in cases where the model predicted a subject referent continuation was not due to the absence of verb-specific associations, but rather because the condition checking prevented these associations from interacting with the general bias to predict pronouns for subject referents. This is why we see ceiling pronoun predictions across all three verb types in cases where the model predicted a subject referent continuation. In other words, our model predicts that implicit causality influences predictions about upcoming referent forms, both for predicted subject referents and for predicted object referents. However, in the case of predicted subject referents the operator implementation prevents the influence of implicit causality from surfacing.

This empirical prediction of our model contrasts with findings from the sentence completion literature which show that implicit causality information does not affect the choice of referring expression (e.g., Rohde and Kehler, 2014; Kehler and Rohde, 2019). Furthermore, this prediction also does not align with the findings of Weatherford and Arnold (2021), who used a novel story re-telling task and found that implicit causality did affect the choice of referring expression. However, they found that participants were more likely to use pronouns for object referents when the verb is object-biased than subject-biased, which is the opposite of what we see in our model's predictions. It remains an open question whether tasks like sentence completion and story re-telling assess the same type of online comprehension processes that we were interested in modeling, as the two tasks require participants to go from being the comprehender to being the producer. Given that our model predicts an interaction that was not present in the input data it was presented with, it is crucial to test this prediction against empirical human data, using a measure that is able to assess uninterrupted online comprehension.

It is again important to note that the model picked up on the main trends in the data, such that the model was much more likely to predict a subject referent continuation for subject-biased verbs and an object referent continuation for object-biased verbs. Furthermore, the model was much more likely to predict pronouns for subject referent continuations and names for object referent continuations. In order to further examine the learning outcomes of the reference learning model, we presented the model with a series of novel items (after the initial learning phase). This revealed that the learned biases generalized to novel contexts. For example, even when the model was unfamiliar with a verb, it still predicted that the discourse would continue about the subject referent above chance level, reflecting a default subject referent continuation bias. Furthermore, in these cases where the model predicted the discourse to continue about the subject referent and was unfamiliar with the verb, it almost exclusively predicted that the subject referent would be in the form of a pronoun, illustrating that in the absence of verb-specific information, form biases are driven by which referent (subject vs. object) the simulated language user anticipates to be rementioned.

The means by which the model was able to learn the different biases was through the use of a domain-general learning mechanism within the PRIMs architecture, known as context-operator learning. Context-operator learning is based on reinforcement learning: the model states signaling a successful strategy or unsuccessful strategy may trigger a positive or negative reward, respectively. When a reward is issued, the associative strengths between the current context and all of the operators that led to the reward are updated. In our model, a reward was issued whenever the model correctly predicted the next referent and the next referent form, which made the model more likely to make the same predictions in similar future contexts. It should be noted that context-operator learning is implemented as reward-based learning because procedural knowledge—which the operators implement—is not available from the environment and, hence, unsupervised learning does not seem to be appropriate. In contrast, the activation of operators and other information in the declarative memory is determined by a sub-symbolic equation that takes into account the frequency and recency of use of this information, which is not reward-based (implementing ACT-R's base-level activation equation; Anderson, 2007). However, as this activation equation seems to be redundant when context-operator learning is applied and did not contribute to our model's behavior, we have turned off base-level activation calculations in our model (but see Juvina et al., 2018 for combining base-level activation with reward-based updating of declarative information, and Hoppe et al., 2022 for a review of supervised declarative learning guided by prediction error). In addition to utilizing this learning mechanism in the PRIMs architecture, we also made other critical modeling assumptions. In what follows, we will discuss these assumptions, along with their implications.

We implemented our model so that it made an explicit prediction about which referent would get referred to, as well as an explicit prediction about the form that referent would take, thereby assuming that language users also make such predictions. The first assumption, that language users anticipate upcoming referents, was directly motivated by the previous literature supporting this idea. For example, Koornneef and Van Berkum (2006) had participants read discourses in which a pronoun followed a clause containing either a subject-biased or an object-biased implicit causality verb (see example (3) in the Background section). The authors found that participants were slower to read these discourses when the gender of the pronoun was inconsistent with the implicit causality bias set up by the verb preceding it. These findings where taken as evidence that language users use implicit causality to anticipate upcoming referents. Similar conclusions have also been drawn in cases where other measures of real-time language comprehension were used, such as visual world eye-tracking (e.g., Pyykkönen and Järvikivi, 2010; Kim and Grüter, 2021) and ERPs (Van Berkum et al., 2007). Most of these findings come from the pronoun resolution literature and therefore involve the processing of specific forms, namely pronouns. However, an empirical prediction of our model is that language users first predict upcoming referents, regardless of the form that gets used to describe them (see Kehler and Rohde's Bayesian model for a similar prediction: Kehler et al., 2008; Rohde and Kehler, 2014; Kehler and Rohde, 2019). Unfortunately, testing this prediction in the context of real-time language comprehension is difficult, given that you cannot refer to a referent without using a specific form. Whether it be a name or a pronoun, specific forms come with their own information and set up their own predictions, which makes it difficult to empirically assess a purely referent-based prediction.

The second assumption, that language users additionally anticipate the form of the upcoming referent, was more indirectly motivated. For example, sentence completion studies show that people are more likely to remention subject referents using pronouns, and similarly are more likely to remention object referents using proper names (e.g., Rohde and Kehler, 2014; Kehler and Rohde, 2019). Furthermore, there is evidence that language users make word-specific predictions during real-time language comprehension (e.g., Wicha et al., 2004; DeLong et al., 2005; Van Berkum et al., 2005). For example, using ERPs, DeLong et al. (2005) found that sentences like “The day was breezy so the boy went outside to fly an airplane” elicit an N400 effect already starting from the indefinite article (as compared to sentences with the more preferred “to fly a kite” ending). This finding was taken as evidence that language users were anticipating the phonological form of the expected word “kite.” However, to our knowledge no study has investigated form-specific referent predictions (i.e., predictions about whether a certain entity will be mentioned using a proper name vs. a pronoun). One study that came close to testing this was that of Featherstone and Sturt (2010). In this study, the authors adapted items from Koornneef and Van Berkum (2006) to include an additional neutral pronoun (i.e., “there”) condition. However, they found no difference in the readings times between this new condition and implicit-causality congruent pronouns, calling into question the nature of word-specific predictions. Next referent form predictions may be something along the lines of “if referent X will be referred to (as I expect), then I expect that form Y will be used,” which is exactly how these predictions were implemented in our model. The fact that our reference learning model was able to pick up on form asymmetries in the input ultimately provides support for the assumption that language users anticipate the form of the upcoming referents, depending on whether that referent was the preceding subject or the preceding object.

One of the primary advantages of cognitive models is that they generate novel predictions that can be tested in (psycholinguistic) experiments. Two more empirical predictions of our model are that language users should be slower to process rementioned subject referents when they are referred to using a name compared to when they are referred to using a pronoun (see Gordon et al., 1993 for the repeated name penalty). Likewise, language users should be slower to process rementioned object referents when they are referred to using a pronoun compared to when they are referred to using a proper name. However, as previously mentioned, it is also important to test whether such effects interact with verb type (subject-biased verbs, object-biased verbs and neutral verbs), as our model predicts this would be the case, which does not align with what is found in the sentence completion literature (see Rohde and Kehler, 2014).

The present study also has implications for current theories of pronoun resolution, which have consistently shown that language users have a preference to interpret ambiguous third-person singular pronouns (like “she”) as referring back to the grammatical subject of the preceding clause. This preference is known as the *subject bias for pronouns* (e.g., Gernsbacher, 1989; Crawley et al., 1990; Arnold et al., 2000). In the context of our reference learning model, this preference of interpreting pronouns as referring back to the grammatical subject could be explained from the idea that language users pick up on asymmetries present in the linguistic input (i.e., that subject referent continuations are more often expressed by using a pronoun), and then apply their knowledge of those asymmetries when processing language in real-time. Although there is no direct evidence of this, different sources of evidence seem to converge on this idea. For example, in a corpus study of children's books, Arnold (1998) found that pronouns co-referred with the subject of the preceding clause 64% of the time. This suggests that within child-directed language input there is a subject bias for pronouns which, according to our model, children should pick up on. Additionally, in a more recent study Arnold et al. (2018) found that participants who scored higher on a measure of written language exposure were more likely to interpret pronouns as referring to the preceding grammatical subject compared to the preceding grammatical object. This suggests that within written (English) language, pronouns more often refer to the grammatical subject of the preceding clause, and that the more exposure language users have, the more likely they are to pick up on this asymmetry and apply it in language processing settings.

Finally, we consider how the meaning and form biases interact with each other during real-time language processing. For this, the visual-world eye-tracking studies that we discussed earlier are relevant. In these studies, participants listened to discourses containing an ambiguous pronoun that is preceded by either a subject-biased or an object-biased implicit causality verb, while at the same time viewing scenes with images of the subject and the object referents [see example (4) in the Background section]. The gaze data in these studies showed an effect of implicit causality even before the onset of the ambiguous pronoun (e.g., Pyykkönen and Järvikivi, 2010; Järvikivi et al., 2017; Kim and Grüter, 2021). Despite this early effect, participants' gaze data still suggested that they ultimately interpreted the pronoun as referring to the preceding subject referent, both for subject-biased verbs and object-biased verbs. Crucially, object-biased implicit causality verbs seemed to simply attenuate the subject interpretation of the pronoun and did not completely flip the interpretation to the object referent. This pattern of results can easily be explained by our model, as when language users encounter an implicit causality verb they likely generate an expectation to hear about the causally implicated referent (driven by an implicit causality bias). In cases where language users expect to hear about a subject referent (i.e., after subject-biased implicit causality verbs), they likely further expect to hear a pronoun, whereas in cases where language users expect to hear about an object referent (i.e., after object-biased implicit causality verbs), they likely do not expect to hear a pronoun (but rather a proper name). Nevertheless, in the visual world eye-tracking experiments, participants were presented with a pronoun in both cases. It seems as though this pronoun itself already signals a subject interpretation (related to the asymmetries in the input). From this it follows that results in these psycholinguistic experiments are driven by two separate biases, one that has to do with which referent will be rementioned, and another one that has to do with how this referent will be mentioned (or in this case, how they will not be mentioned).

The present study serves as a first step in using cognitive modeling to simulate the learning and use of reference biases. As such, we made certain choices that could be explored in the future. For example, as previously mentioned, the reference asymmetries in the input data were inspired by patterns found in the sentence completion literature. However, because it is currently unknown what distributions exist in actual language input to language learners, it is important for future modeling studies to explore different possible distributions, including unbalanced distributions where there is an interaction between continued referents and their forms. Furthermore, all cognitive architectures place their own constraints on how models can be implemented. For example, in order to make use of PRIMs' context-operator learning we could only issue a single reward, such that rewards were only issued in cases where the model accurately predicted the upcoming referent and the referent form. This contributed to verbs having a positive association with form operators, despite there being no association between the implicit causality of a verb and the likelihood of using a particular form in the input data. Without this constraint placed upon us by the architecture, another possibility would have been to receive a partial reward for being partially correct, which could influence the learning outcomes of the model. This highlights the fact that cognitive modeling helps us pinpoint several important questions that often get overlooked when discussing strictly experimental data.

In sum, we were able to show that seemingly complex linguistic behavior can be explained by domain-general cognitive learning and processing mechanisms. In particular, our cognitive model was able to learn the implicit causality bias from asymmetries in the linguistic input. This result was argued to have implications for accounts of the implicit causality bias and theories of pronoun resolution. Furthermore, based on the output of our cognitive model, novel predictions were generated about referring expressions and their meanings that can be tested in psycholinguistic experiments. Although we chose to explore these particular biases, the same method could be applied to help explain how we acquire other linguistic knowledge and how that knowledge comes into play during real-time language processing, for example, reference biases related to animacy. We believe that there are many advantages of studying language using domain-general architectures, as the models developed in these architectures may generate more cognitively plausible and specified theories of language, situated within general human cognition.

## Data availability statement

The PRIMs model code, as well as the scripts used to generate the input data and analysis plots are available online at https://git.lwp.rug.nl/p251653/learning-reference-biases.

## Author contributions

AT, PH, NT, and JvR conceived of the research and were involved in the interpretation of results. AT implemented the model and wrote the first draft of the manuscript. PH, NT, and JvR provided feedback on the manuscript. All authors contributed to the article and approved the submitted version.

## Funding

This research was supported by a Veni grant from the Netherlands Organization for Scientific Research NWO (No. 275-70-044, JvR).

## Conflict of interest

The authors declare that the research was conducted in the absence of any commercial or financial relationships that could be construed as a potential conflict of interest.

## Publisher's note

All claims expressed in this article are solely those of the authors and do not necessarily represent those of their affiliated organizations, or those of the publisher, the editors and the reviewers. Any product that may be evaluated in this article, or claim that may be made by its manufacturer, is not guaranteed or endorsed by the publisher.
